# The effectiveness and safety of heat/cold therapy in adults with lymphoedema: systematic review

**DOI:** 10.1080/09638288.2023.2231842

**Published:** 2023-07-10

**Authors:** J. E. Hill, J. C. Whitaker, N. Sharafi, O. Hamer, A. Chohan, C. Harris, A. Clegg

**Affiliations:** a Synthesis, Economic Evaluation and Decision Science (SEEDS) Group, University of Central Lancashire, Preston, UK; b Allied Health Research Unit, University of Central Lancashire, Preston, UK

**Keywords:** Lymphoedema, heat therapy, cold therapy, limb circumference, limb volume

## Abstract

**Purpose:**

The aim of this review is to assess the efficacy and safety of using heat and cold therapy for adults with lymphoedema.

**Methods:**

A multi-database search was undertaken. Only studies which included adults with lymphoedema who were treated with heat or cold therapy reporting any outcome were included. Screening, data extraction, and assessment of bias were undertaken by a single reviewer and verified by a second. Due to the substantial heterogeneity, a descriptive synthesis was undertaken.

**Results:**

Eighteen studies were included. All nine studies which assessed the effects of heat-therapy on changes in limb circumference reported a point estimate indicating some reduction from baseline to end of study. Similarly, the five studies evaluating the use of heat-therapy on limb volume demonstrated a reduction in limb volume from baseline to end-of-study. Only four studies reported adverse events of which all were deemed to be minor. Only two studies explored the effects of cold therapy on lymphoedema.

**Conclusions:**

Tentative evidence suggests heat-therapy may have some benefit in treating lymphoedema with minimal side effects. However, further high-quality randomised controlled trials are required, with a particular focus on moderating factors and assessment of adverse events.

**Implications for rehabilitation**
- This review highlights the potential benefit that heat therapy may have on reducing limb circumference and volume for adults with lymphoedema.
- There was no evidence that controlled localised heat therapy was unsafe.
- The current evidence-base is at a point where no specific clinical recommendations can be made.
- The use of heat therapy should only be applied as part of a methodologically robust study to treat lymphoedema.

## Introduction

Lymphoedema is a chronic disease that occurs when the lymphatic system fails, resulting in the accumulation of excess fluid in the body’s tissue [1]. Approximately, 140 million to 250 million people worldwide have a form of lymphoedema, although this is considered an underestimate [2,3]. Lymphoedema typically presents as either primary or secondary lymphoedema [1]. Primary lymphedema is a rare inherited or congenital condition that causes a malformation of the lymphatics system, more often because of genetic mutation [1,4]. Secondary lymphoedema is most common and typically results from an injury, or obstruction to the lymphatic system [1,4,5]. The causes of lymphoedema are wide-ranging but are often the result of obstructive lesions (e.g., tumour) within the lymphatic system, infections, and complications of surgical procedures [1]. Globally, the single primary cause of lymphoedema is nematode infection (known as filariasis), which, despite recent advances, is associated with more than 16 million cases worldwide [6].

Once diagnosed and classified, the treatment and management of lymphoedema begins with conservative strategies [7]. Conservative treatments typically involve an intensive phase of treatment known as decongestive lymphatic therapy (DLT) and consists of centripetal massage of the lymphatics, manual lymph drainage (MLD) [8], multi-layer lymphoedema bandaging (MLLB) [9], and the use of pneumatic compression devices followed by a maintenance phase, which supports the patient to self-manage their condition with compression garments, exercise, skin hygiene, and care [10,11]. Surgical approaches involve restoring lymphatic flow to the limb (either by reconstruction of lymphatic channels or by bridging lymphoedematous tissue with normal lymphatics), or removing excess tissue to reduce the limb to a functional size (skin grafting or subcutaneous excision) [12]. For lymphedema treatments to be effective, early diagnosis, treatment, and patient compliance are necessary [13].

Literature and international guidelines suggest that patients should take preventative measures which can help reduce lymphedema symptoms and prevent complications [1,13,14]. Some of these preventative strategies include, avoiding limb constriction, needles, air travel, vigorous exercise, and extreme temperatures [15,16]. Although many of these strategies are evidence based, guidance on the effects of temperature appears less certain given the conflicting evidence on the risks posed [17–19]. Historically, research has recommended that patients avoid prolonged exposure to heat and cold, as it is thought that it may increase blood flow, intensifying lymphatic load [14,15,20]. In contrast, recent evidence shows a beneficial effect, with heat exposure reducing lymphedema [17]. Given the uncertainties, we conducted a systematic review of the evidence to assess the effectiveness of heat and cold therapy for adults with lymphoedema.

## Aims

The aim of this review was to assess the efficacy and safety of using heat and cold therapy for adults with lymphoedema.

## Design and methods

Prior to commencing this systematic review, a protocol was registered on Prospero (registration number: CRD42022309475). This systematic review has been reported in accordance with Reporting Items for Systematic Reviews and Meta-Analyses [21].

### Searches

The following electronic bibliographic databases were searched on 22 March 2023: MEDLINE (Ovid), Embase (Ovid), CINAHL Complete (EBSCOhost), AMED (EBSCOhost), Cochrane Library via Wiley (all databases), and Web of Science (indexes: SCI-EXPANDED; SSCI; AHCI; CPCI-S; CPCI-SSH; ESCI) using the terms identified by the review team (see Appendix 1 for full search strategies for each database). No language or other limits were applied to the searches. Additional snowball sampling of all included studies was undertaken. Duplicate removal was undertaken using EndNote (version 9.3) and then in Rayyan [22].

### Study selection

Only uncontrolled before-and-after studies, controlled trials both randomised and nonrandomised were included. Studies had to include adults with primary or secondary lymphoedema defined by the author from any clinical setting. The intervention had to use heat or cold therapy (thermotherapy or cryotherapy) using any modality with no minimum or maximum number of exposures (e.g., full, or partial body cryotherapy, cold water, ice packs, heat packs, hot water, ultrasound, microwave, and laser therapy). Low level laser therapy was excluded as producing heat is not its primary purpose. No comparator was specified (e.g., usual care, placebo, exercise, massage/physiotherapy, MLD, compression bandages/garments, sequential pneumatic compression, etc.). No specified inclusion criteria were set for outcome of included studies.

### Data extraction and quality assessment

Abstract and title screening were carried out by a single reviewer using Rayyan (NS and JH). This selection process was piloted with 10% of abstracts and titles being screened by a second reviewer independently (JW). A Kappa score was calculated for this piloted screening process. Substantial agreement (0.61–0.80) was required before continuation. If the agreement level was unable to be achieved, this process was repeated until substantial agreement was achieved.

Full paper screening was carried out by a single reviewer and verified by a second reviewer (NS & JW or JH & OH). Reasons for exclusion were recorded and reported for full paper screening. Data extraction was undertaken by a single reviewer and verified by a second reviewer using a pre-piloted form (NS, JW, or JH). Discrepancies within abstract and title, full paper screening and data extraction were resolved by discussion, if consensus was unable to be achieved arbitration was carried out by a third reviewer (JH and JW).

The data items extracted were: study type, population description, condition, clinical setting, country, age, gender, location of lymphoedema, lymphoedema type, time of diagnosis, intervention duration, temperature, mode of heat or cold, control group description, all outcomes, number of participants and number of adverse events, adverse event type, number of patients as a ratio completed treatment, conflicts of interest and funding.

### Quality assessment

Study level quality assessment was undertaken by a single reviewer and verified by a second reviewer (NS, JH, or JW). Discrepancies within the quality assessment were resolved by discussion, if consensus was unable to be achieved, arbitration was carried out by a third reviewer (JH or JW). Depending on the study design, either the methodological index for non-randomized studies (comparative or non-comparative) (MINORS) [23] or the randomised control trial risk of bias in randomized trials (RoB-1) was used [24]. Both tools were selected due to them being deemed to be valid, reliable [23,25] and the review team having experience of using the tools.

### Data synthesis

Due to the expected wide variation in study design, interventions, and outcomes, a narrative synthesis approach was used to assess effectiveness and types of adverse events. Study findings were clustered around two broad categories of interventions of heat and cold therapies and sub-sectioned into varying modalities. Due to the wide variation in outcome type, comparison of outcomes and unit of measurement, a “vote counting” method was applied. This approach was used as it was felt that pooling estimates of individual studies may be misleading due to the inherent heterogeneity [24]. For each modality, the number of positive studies for every outcome and the number of studies reporting a statistically significant positive outcome (*p* ≥ 0.05) were reported. A positive study was classified as an improvement in the outcome being measured from baseline to end of study within the intervention group. For the interpretation of the findings, the number of positive studies was the primary measure. We did not interpret the number of studies demonstrating a statistically significant difference as an indication of degree of improvement, rather that we reported it as an indication of the probability of the improvement occurring by chance within these individual studies. Any comparisons between groups were described using a similar method. Therefore, these findings will answer the question if there is any evidence of efficacy rather than giving a specific estimate of efficacy [26].

Acceptability of treatment for heat and cold therapies was meta-analysed using a random effects model (DerSimonian–Laird) of the proportion of people who completed the intervention (number of participants reported in the results section) compared to those who started the study (number of participants reported in methods) [27]. Heterogeneity was assessed through visual inspection of forest plots and the *I*-squared statistic [24]. Meta-analysis was undertaken using OpenMeta [Analyst] [28].

## Results

### Study identification and characteristics

**Figure 1.**
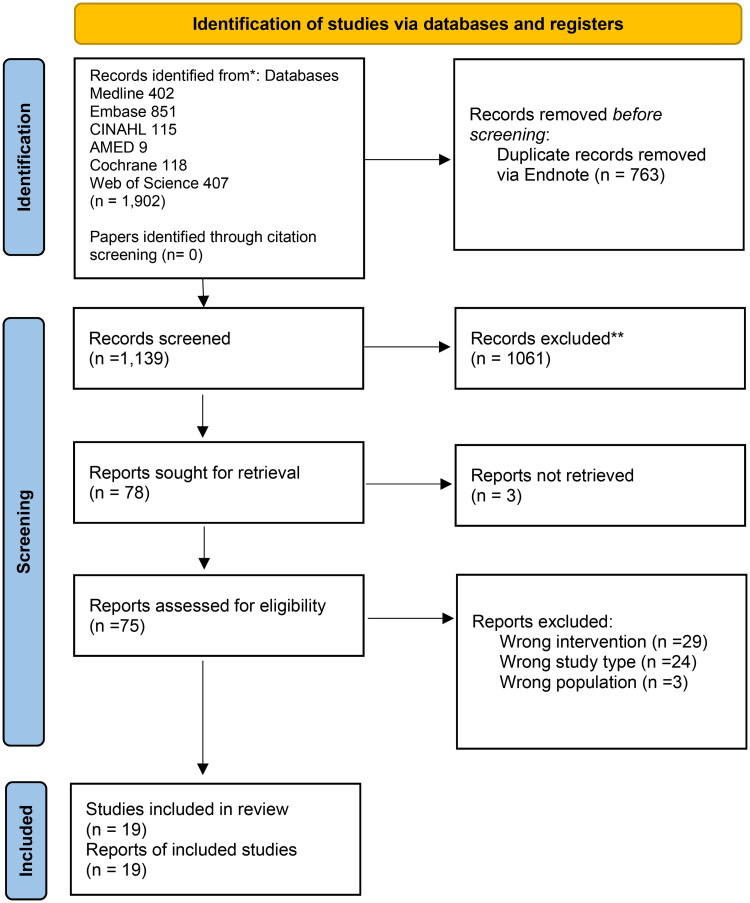
PRISMA flow diagram.

After duplicate removal, 1139 citations were identified (see Figure 1 for full paper screening and reasons for exclusion). A Kappa score of 0.75 (substantial agreement) with 97% agreement was achieved during abstract and title screening between the two reviewers using a 10% sample. After title and abstract screening, 78 full papers were retrieved, of which 18 studies were included (19 citations). Of these 19 included studies, 11 studies used a before-and-after design [18,19,29–37], four used a controlled before-and-after study design [38–41], three were randomised controlled trials (RCTs) [41–43], and one study used a cross-over randomized study design [44]. They were published between 1986 and 2022 [37,43]. These studies took place in various countries, of which seven were from China [30,32,34,36,37,40,41], four from Italy [19,29,31,38], three studies from Japan [35,42,45], two studies from India [18,39], and one study each from Brazil, Egypt, and USA [33,43,44].

Across the 18 included studies, a total of 1137 people with lymphoedema were included (see Table 1 for study characteristics). The majority of studies included adults with secondary lymphoedema of the legs [35,36,40,41,45], arms [32,33,38,43], or both arms and legs [19,29,30]. Included studies took place in a range of clinical environments of which nine were within a hospital [32,34–37,39–42], six in outpatients [30,31,33,38,43,45], three in a lab [19,29,44], and one at home [18]. Three studies included a mixed adult sample [30,34,37]. Out of the 18 included studies, only two studies assessed the effectiveness of cold therapy on lymphoedema using ethanol–water [33] and cold air therapy [43]. One study aimed to reduce the surface temperature of the skin between 32.4 and 33.8 °C [33]. One study did not report exact skin temperature but stated using cold air at a temperature of −32 °C [43].

**Table 1.** Study characteristics.

Author	Country	Study type	Clinical setting	Location of lymphoedema	Lymphoedema type	Mean age (years)	Gender (female %)	Temperature of tissue (°C)	Heat or cold	Modality of treatment
Askary and Elshazly [43]	Egypt	RCT	Outpatients	Arms	Secondary	49	100%	N/R	Cold	Cold air therapy
Balzarini et al. [38]	Italy	Controlled before-and-after study	Outpatients	Arms	Secondary	N/R	100%	N/R	Heat	Ultrasound
Campisi et al. [19]	Italy	Before-and-after study	Lab	Mix	Secondary	26	0.95	41 °C	Heat	Hot water
Campisi et al. [29]	Italy	Before-and-after study	Lab	Mix	Secondary	36	100%	41 °C	Heat	Hot water
Cao et al. [30]	China	Before-and-after study	Outpatients	Mix	Secondary	8–72	N/R	N/R	Heat	Microwave + bandaging
Chang et al. [41]	China	RCT	Hospital	Legs	Secondary	36.7	0.62	N/R	Heat	Microwave
Chang et al. [34]	China	Before-and-after study	Hospital	Legs	Primary	7–63	51	39–41 °C	Heat	Microwave
Fox et al. [31]	Italy	Before-and-after study	Outpatients	Mix	Both	43	67%	38 °C	Heat	Electromagnetic
Gan et al. [32]	China	Before-and-after study	Hospital	Arms	Secondary	N/R	100%	N/R	Heat	Microwave
Gogia et al. [39]	India	Controlled before-and-after study	Hospital	Legs	N/R	N/R	N/R	N/R	Heat	Light therapy OR light therapy + interferential therapy OR light therapy + interferential therapy + compression
Hasegawa and Ohkuma [45]	Japan	Controlled before-and-after study	Outpatients	Legs	Secondary	66	88%	N/R	Heat	Microwave + vibration + bandaging
Kulkarni et al. [18]	India	Before-and-after study	Home	N/R	Secondary	N/R	N/R	45–50 °C	Heat	Light therapy
Li et al. [17,36]	China	Before-and-after study	Hospital	Legs	Secondary	N/R	N/R	42 °C	Heat	Infrared
Liu and Olszewski [40]	China	Controlled before-and-after study	Hospital	Legs	Secondary	N/R	N/R	39–44 °C	Heat	Microwave or hot water
Mariana et al. [44]	Brazil	Randomised crossover study	Lab	Legs	N/R	48.5	71%	37.3–40.2 °C	Heat	Electric blanket + mechanical lymph drainage
Mayrovitz and Yzer [33]	USA	Before-and-after study	Outpatients	Arms	Secondary	61	100%	32 °C	Cold	Ethanol–water
Ohkuma [42]	Japan	RCT	Hospital	Mix	Both	N/R	N/R	N/R	Heat	Microwave + bandaging
Ohkuma [35]	Japan	Before-and-after study	Hospital	Legs	Secondary	35–76	100%	43 °C or 50 °C	Heat	Microwave + Vibration + bandaging
Zhang et al. [37]	China	Before-and-after study	Hospital	Mix	Both	7–77	53%	39–40 °C	Heat	Microwave

Mix: lymphoedema of the legs and arms; both: primary and secondary; +: additional treatment; RCT: randomised controlled trial; N/R: not reported in study; Lab: laboratory.

The remaining 17 studies used localised heat therapy provided through 20 modalities, of which five studies used microwave therapy [32,34,37,40,41]; four studies used microwave plus bandaging [30,42]; with two including vibration [40,45]; three studies used hot water [19,29,40]; two studies used light therapy [18,39]; one study used light therapy plus interferential therapy [39]; light therapy plus interferential therapy plus compression [39]; infrared [36]; electric blanket plus mechanical lymph drainage [44]; ultrasound [38] and electromagnetic therapy [31]. The majority of these studies that reported skin temperature set a target tissue temperature of between 39 and 42 °C [19,29,31,34,36,37] and the majority of studies which reported total intervention treatment time provided treatments between 1200 up to 3600 min of treatment [19,34–36,40–42].

### Quality assessment

Nine out of 11 before-and-after studies were deemed to be of low quality (overall score less than 13) [18,29–32,34–37] (see Tables 2 and 3 for quality assessment of all included studies). With the three main issues being lack of blinding of endpoints [18,29–32,34,36], prospective calculation of the sample size [18,30,32,34–37] and appropriate follow-up period [18,29,31,34–36]. One out of the four non-randomized controlled trials was judged to be of low quality (less than 16) [45]. With the main issues being a lack of blinding of endpoints [38,39,45], inappropriate control group [19,39,45], and lack of clarity regarding if the control and an intervention group were treated at the same time [19,39,45]. Three out of the four RCTs were judged to be at high risk of bias [35,43,44]. All four RCTs had methodological limitations with randomisation sequence generation, lack of allocation concealment and selective reporting bias due to the lack of protocol registration [41–44]. The majority of issues within the before and after studies, randomised and non-randomised studies were caused by poor reporting standards.

**Table 2.** Quality assessment using methodological index for non-randomized studies (comparative or non-comparative).

Author	1. A clearly stated aim	2. Inclusion of consecutive patients	3. Prospective collection of data	4. Endpoints appropriate to the aim of the study	5. Unbiased assessment of the study endpoint	6. Follow-up period appropriate to the aim of the study	7. Loss to follow up less than 5%	8. Prospective calculation of the study size:	9. An adequate control group	10. Contemporary groups	11. Baseline equivalence of groups	12. Adequate statistical analyses	Total score
Balzarini et al. [38]	2	2	2	2	0	2	2	0	0	2	0	2	16/24
Campisi et al. [29]	0	2	0	0	0	0	0	2	N/A	N/A	N/A	N/A	4/16
Campisi et al. [19]	0	2	2	0	2	0	2	2	0	0	2	2	14/16
Cao et al. [30]	2	2	2	1	0	2	2	0	N/A	N/A	N/A	N/A	11/16
Chang et al. [34]	0	0	0	0	0	0	2	0	N/A	N/A	N/A	N/A	2/16
Fox et al. [31]	2	0	1	2	0	0	2	1	N/A	N/A	N/A	N/A	8/16
Gan et al. [32]	2	2	2	2	0	2	2	0	N/A	N/A	N/A	N/A	12/16
Gogia et al. [39]	2	2	2	2	0	2	2	2	0	0	2	2	18/24
Hasegawa and Ohkuma [45]	2	2	2	2	0	0	0	0	0	0	2	2	12/24
Kulkarni et al. [18]	1	1	2	1	0	0	2	0	N/A	N/A	N/A	N/A	7/16
Li et al. [17,36]	2	0	2	2	0	0	0	0	N/A	N/A	N/A	N/A	6/16
Liu and Olszewski [40]	2	2	2	2	2	2	2	0	2	2	2	2	22/24
Mayrovitz and Yzer [33]	2	2	2	2	2	2	2	2	N/A	N/A	N/A	N/A	16/16
Ohkuma [35]	2	2	2	2	0	0	2	0	N/A	N/A	N/A	N/A	10/16
Zhang et al. [37]	2	2	2	2	2	2	1	0	N/A	N/A	N/A	N/A	13/16

0: not reported; 1: reported but inadequate; 2: reported and adequate; N/R: not applicable; N/A: not applicable.

**Table 3.** Risk of bias of randomised controlled trials.

Author	Random sequence generation (selection bias)	Allocation concealment (selection bias)	Blinding (performance and detection bias) participants	Blinding (performance and detection bias) those delivering the intervention	Incomplete outcome data (attrition bias)	Selective reporting (reporting bias)	Other biases	Overall risk of bias
Askary and Elshazly [43]	Unclear	Unclear	Unclear	Unclear	Unclear	Unclear	Low	Unclear
Chang et al. [41]	High	Unclear	Low	Low	Low	Unclear	Low	Low
Ohkuma [42]	Unclear	Unclear	High	Unclear	Low	Unclear	Low	High
Mariana et al. [44]	Unclear	Unclear	Unclear	Unclear	Low	Unclear	Low	High

### Microwave therapy

Five studies assessed the effectiveness of microwave therapy, with one RCT (low risk of bias) and non-RCT (quality score (QS): 22/24) comparing microwave therapy against placebo [41] and hot water therapy [40], respectively, and three before-and-after studies (QS: 2, 12, 13/16) [32,34,37]. The one RCT found a statistically significant difference in the proportion of patients, who reported an improvement in feeling of swelling and restricted mobility after the intervention period compared to those who received placebo [41]. There was also a nonsignificant difference in the proportion of patients who reported an improvement in burning pain or feeling heavy, compared to the placebo group. In the non-RCT study, there was a greater improvement in circumference in microwave therapy compared to hot water therapy; however, this was reversed for limb volume [40] (See Table 4 for full results).

**Table 4.** Results for effect of heat/cold therapy from baseline to end of study in intervention group.

Author	Modality	Outcomes/effect																			
		1		2		3		4		5		6		7		8		9		10	
Askary and Elshazly [43]	Cold air therapy	Limb circumference (wrist)	^b^	Limb circumference (below elbow)	^b^	Limb circumference (above elbow)	^b^	Thickness	^b^												
Balzarini et al. [38]	Ultrasound	Limb volume	^a^	Subjective assessment of firmness	^a^																
Campisi et al. [19]	Hot water	Limb circumference	N/R	Limb volume	^a^	Partial recovery (1 year)	^a^														
Campisi et al. [29]	Hot water	Limb circumference	N/R	Lymph flow	N/R	Limb oedema	N/R														
Cao et al. [30]	Microwave + bandaging	Limb circumference	^b^	Tissue tonicity	^b^	ABC immune-histochemistry	^b^														
Chang et al. [41]	Microwave	Limb circumference	^a^	Limb volume	^b^	Tonometry	^b^	Feeling of swelling	^b^	Burning pain	^a^	Feeling of heaviness	^a^	Restricted mobility	^b^	Quality of life (VAS)	^b^	IL-18	^b^	TGF-b1	^b^
Chang et al. [34]	Microwave	Limb circumference	^b^	Limb volume	^b^																
Fox et al. [31]	Electromagnetic	Circumference limb	^a^	Lymphangitis attacks	^a^																
Gan et al. [32]	Microwave	Limb circumference	^b^	Limb volumetric changes	^b^	Erysipelas attacks	^b^	Tonometry	^b^												
Gogia et al. [39]	Light therapy OR light therapy + interferential therapy OR Light therapy + interferential therapy + compression	Limb circumference (all groups)	^a^																		
Hasegawa and Ohkuma [45]	Microwave + vibration + bandaging	Calcitonin gene-related peptide levels	^b^																		
Kulkarni et al. [18]	Light therapy	Limb circumference	^a^																		
Li et al. [17,36]	Infrared	Frequency dermatolymphangio-adenitis	^b^	Tightness (VAS)	^b^	Heaviness (VAS)	^b^	Solidity (VAS)	^b^	Pain (VAS)	^b^	Discomfort (VAS)	^b^	Numbness (VAS)	^b^						
Liu and Olszewski [40]	Microwave or hot water	Limb circumference (both groups)	^b^	Limb volume (both groups)	^b^																
Mariana et al. [44]	Electric blanket + mechanical lymph drainage	Limb volume	^a^																		
Mayrovitz and Yzer [33]	Ethanol–water	Limb circumference	N/R	Indentation force (indentation forces to 1.3 mm)	^b^	Indentation force (indentation forces to 4.0 mm	^b^	Tissue dielectric constant	^a^												
Ohkuma [42]	Microwave + bandaging	Limb volume	^a^																		
Ohkuma [35]	Microwave + vibration + bandaging	Limb circumference	^a^																		
Zhang et al. [37]	Microwave	Limb circumference	^a^	Limb volume	^a^	Erysipelas attacks	^b^														

+: additional treatment; VAS: visual analogue scale; N/A: not applicable.

^a^ A positive improvement from baseline to end of study.

^b^ Eight statistically significant improvement from baseline to end of study (*p* = 0.05).

All five studies that used microwave therapy demonstrated a positive improvement in the intervention group from baseline to end of study for limb circumference [32,34,37,40,41] of which three were statistically significant [32,34,40]. Similarly, all five studies demonstrated a positive improvement in limb volume comparing baseline to end of study [32,34,37,40,41] of which four were statistically significant [32,34,40,41]. Two studies found a statistically significant improvement for both tonometry [32,41] and the number of erysipelas attacks [32,37]. One study found an improvement in subjective reporting of burning pain, feeling of heaviness and a statistically significant improvement in restricted mobility of the affected limb from baseline to end of study [41].

### Microwave therapy plus

Four studies assessed the effectiveness of microwave therapy plus additional treatment, with one RCT (high risk of bias) [42] and three before-and-after studies (QS: 11, 10, 12/16) [30,35,45]. One RCT found a statistically significant difference in limb volume between microwave and bandages compared to bandages or microwave therapy alone [42].

When microwave therapy was combined with bandaging, there was a positive improvement in the intervention group in two studies for limb circumference [30] and limb volume [42] from baseline to end of study, of which limb circumference was statistically significantly improved in one study [30]. There was also a statistically significant improvement in tissue tonicity and ABC immunohistochemistry [30].

The two before-and-after studies which used microwave therapy plus vibration plus bandages found a positive improvement for limb circumference [35] and a statistical significant improvement in calcitonin gene-related peptide levels from baseline to end of study [45].

### Hot water

Out of the three studies that reported using hot water therapy [19,29,40], only non-RCT (QS: 22/24) and one before and after study (QS: 14/16) reported outcomes [19,40]. Of these two studies, one found a positive improvement [19] and the other study found a statistically significant improvement in limb volume from baseline to end of study in the intervention group [40]. One study also found that at one year, there was no difference between primary, secondary, and acute lymphoedema recovery rates [19].

### Light therapy and infrared

The effects of light (heat) therapy were assessed in a non-RCT (QS: 18/24) [39], which compared light therapy with combinations of either light therapy and interferential therapy or light therapy and interferential therapy plus compression, and two before-and-after studies of light therapy (QS: 7/16) [18] or infra-red therapy, respectively (QS: 6/16) [36]. Of these studies, the non-RCT found positive improvement in limb circumference for light therapy plus interferential therapy and combination (light therapy + interferential therapy + compression). When the interventions were compared, there was a statistically significant improvement in total limb circumference reduction at end of study in the compression group (bandage compression) compared to the light therapy group and the combination group [39]. There was a greater improvement in total limb circumference in the light therapy group compared to combination and light therapy plus interferential therapy group [39].

Two out of two studies found a positive improvement in limb circumference for light therapy alone from baseline to end of study in the intervention group [18,39]. Only the before-and-after study used infrared therapy and found a statistically significant improvement in patient subjective reporting of tightness, heaviness, solidity, pain, discomfort, numbness, quality of life, and frequency of lymphedema accompanied with dermatolymphangioadenitis from baseline to end of study [36].

### Electromagnetic resonance, electric blanket, and ultrasound

One out of one randomised crossover study found a positive improvement for electric blanket heat treatment plus mechanical lymph drainage for limb volume comparing baseline to end of study (high risk of bias) [44]. However, there was no statistically significant improvement in limb volume when treated with electric blanket heat treatment plus mechanical lymph drainage compared to mechanical lymph drainage alone [44].

One out of one non-randomised control study found an improvement in limb volume and subjective assessment of firmness comparing baseline to end of study (QS: 16/24) [38]. There was a statistically significant improvement in limb volume from baseline to 12 months when comparing the ultrasound therapy group to the mechanical pressure therapy group [38]. There was also a greater reduction in subjective pain at 12 months in the ultrasound group compared to the mechanical pressure therapy group [38].

One out of one study found when using electromagnetic resonance therapy, there was an improvement in limb circumference and number of lymphangitis attacks (attacks per year) (QS: 8/16) [31].

### Ethanol–water (cold therapy)

One RCT and one before and after study examined the effect of cold therapy using cold air [43] or ethanol–water [33]. The RCT found a statistically significant difference between the cold air therapy group compared to usual care, for thickness of epidermis and dermis and circumference of the wrist, above and below the elbow at 6 weeks and 12 weeks of treatment (high risk of bias) [43]. The before and after study using ethanol–water found a statistically significant reduction in skin tissue hardness at levels 1.3 mm and 4 mm when comparing baseline to end of study (QS: 16/16) [33]. Tissue dielectric constant (TDC) used to measure skin water level, also had a positive improvement from baseline to end of study [33].

### Attrition rate of intervention group

**Figure 2.**
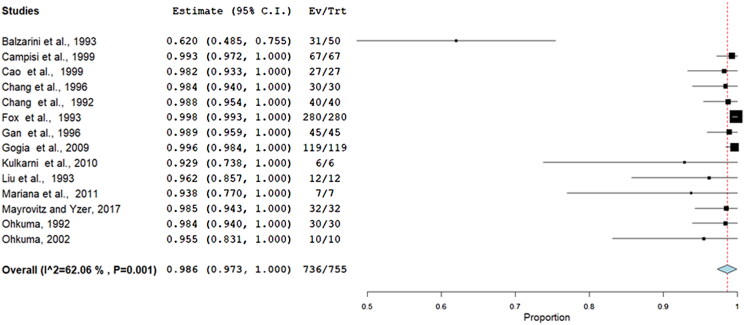
Meta-analysis of attrition rates of treatment.

A random effects meta-analysis found the completion rate to be 99% (95% confidence interval 97–100%) of studies, which reported both data points. Only one study had a less than a hundred percent completion rate. There was moderate statistically significant heterogeneity (*I*^2^ = –62%). See Figure 2, for the forest plot.

### Adverse events

Only four studies out of the 18 included studies reported adverse events [35,38,42,45]. One study using electromagnetic resonance treatment noted that a “few patients” out of 16 participants reported having a slight headache after treatment [45]. A study using microwave therapy plus bandages reported that two patients out of 30 reported lymph node swelling and cellulitis [42]. In one study using microwave plus vibration and bandaging, one patient out of nine developed transient skin erythema [35]. Another study reported reasons for noncompletion across all interventions studied (i.e., 150 participants received either ultrasound, ultrasound plus elastic sleeve, mechanical pressure, or mechanical pressure plus elastic sleeve), with 12 patients having recurrence of cancer and 42 reporting non-compliance of treatment [38].

## Discussion

With no cure for lymphoedema, attention has focused on conservative management approaches to managing its symptoms, specifically through improving the flow of fluid through the lymphatic system and preventing its build-up [46]. Guidance has recommended the wearing of active or passive compression garments, multilayer bandaging, exercise, and specialised massage techniques (e.g., manual lymphatic drainage) to help promote the drainage of fluid and prevent it accumulating in the body [47]. The importance of good skin care to prevent infection is emphasised [47]. Surgery to debulk tissue through liposuction and lymphatic reconstruction through bypass provide other options [47]. Although temperature-based treatments have been used with the intention of improving drainage of lymphatic fluid [48], current clinical advice given to people with lymphoedema indicates that extremes of temperature should be avoided [16,49–51]. It is thought that such extremes may increase blood flow and increase lymphatic load [15]. Uncertainties around possible treatment options cause concern for people with lymphoedema and for those providing treatment who want to receive and provide the most effective care [52].

In systematically reviewing the evidence on the use of “hot” and “cold” therapies for managing lymphoedema, it was evident that there were some benefits, particularly in reducing limb volume. Approaches that applied heat, whether through microwave therapy alone [32,34,37,40,41] or in combination with bandages [30] and bandages plus vibration [35] through hot water treatment [19,29,40], electromagnetic resonance [31], and light therapy alone [18], and in combination with interferential therapy and/or compression [39] showed benefit in reducing limb circumference. Similar benefits from heating were found on limb volume following the use of microwave [32,34,37,38,41], microwave with bandaging [42], hot water [19], ultrasound [38], and electric blankets combined with mechanical lymph node drainage [44]. Measures of indentation force (i.e., skin firmness) improved following the use of heat through the use of microwave [32,41] and microwave plus bandaging [30]. Benefits following heat therapy were found on other symptoms of lymphoedema and subjective measures of quality of life [19,30–33,36,37,41,45]. Cold therapy, through the use of ethanol–water or colder air, had beneficial effects on indentation force (i.e., skin firmness), TDC (measure of tissue water), skin thickness, and limb circumference [33,43]. Adverse events were rarely reported in studies, with those identified including headache [45], lymph node swelling [42], cellulitis [42], and transient skin erythema [35]. Although non-compliance with ultrasound, ultrasound plus elastic sleeve, mechanical pressure, or mechanical pressure plus elastic sleeve were reported [38], attrition from studies was low.

The benefits of “hot” and “cold” therapies were predominantly shown in studies conducted under specific treatment conditions, including controlled temperature ranges (39–42 °C) [19,29,31,34,36,37], prolonged treatment periods (1200–3600 min) [19,34–36,40–42] and within clinical environments, laboratory [19,29,44]; hospital [32,34–37,39–42]; outpatient [30,31,38,45]. It is unclear whether the suggested benefits would be maintained outside these specific conditions, an important consideration given the long-term goal of self-management at home [47]. Importantly, the findings should be interpreted with caution given the varied study designs, their methodological quality and the risk of bias. Only two before-and-after studies [33,37], three non-RCTs [38,39] and one RCT [41] were at low risk of bias, which may have influenced the outcomes reported. With limited comparative studies, it is unclear if the “hot” and “cold” therapies are more or less effective than other treatment options.

In other areas of rehabilitation, the evidence on the benefits of controlled heat and cold exposure for chronic swelling is inconsistent [53–57]. Subsequently making it difficult to make a global recommendation for heat and cold to reduce swelling in all clinical scenarios. The reviews in this area are either very specific to a treatment type [57] or to a specific condition [58,59]. But overall, there is a notable lack of systematic reviews exploring the effect of heat and cold therapy on swelling. When using ultrasound as a heat source, a previous Cochrane review found no evidence that ultrasound reduced swelling compared to sham ultrasound [57]. This was based upon only three low quality random controlled trials.

The findings of this review are consistent with this previous Cochrane review in that there was limited evidence to support the benefit effects of ultrasound on swelling [38]. Regarding other chronic conditions where swelling is common, cryotherapy has shown to help with reducing localised swelling when applied in both patients with rheumatoid arthritis and osteoarthritis [58,59]. However, like this review the evidence is limited and methodologically weak. It is important to note that all three reviews are now at least two decades old and the evidence underpinning this effect may have substantially changed.

### Strengths and limitations

This systematic review has certain strengths and limitations that should be taken into consideration when interpreting the findings of this review. The strengths of this systematic review are: that it was registered on Prospero prior to commencing the review; no post hoc amendments were made; a multi-database search was used [60] with citation screening (no additional papers were identified) [61] and a pre-piloted form was used for data extraction [62].

The main limitations of this review were that abstract and title screening was undertaken by a single reviewer with a piloting process [63]. However, substantial agreement was able to be achieved within the 10% piloting process (Cohen kappa score). Similarly, full paper screening, data extraction, and assessment of bias were undertaken by a single reviewer and verified by a second reviewer but not independently. A further limitation was that three papers were unable to be retrieved for full paper screening. An email was sent to the corresponding authors but there was no response. A further request was made to the British library for these articles; however, they were unable to locate them. Nevertheless, these three studies were not identified to be included studies, rather that they were studies which needed additional information to make a more informed decision at full paper screening. Due to the wide heterogeneity of studies and outcome measures, a “vote counting” method was employed. This method does not produce an estimate of effect and does not take into account the individual weighting of studies [26]. This method also does not consider if the difference was statistically significant [26]. Within this review, the number of statistically significant studies was reported but this was not interpreted as an indication of direction, rather that it was reported as an indication of the probability of the improvement occurring by chance within these individual studies. Furthermore, when using vote counting other such principles, such as imprecision, inconsistency, and publication bias are difficult to assess, which is typically needed to establish a certainty in a directional effect. It is important to note that due to the before and after data collection utilised in the majority of studies within this review, that a meta-analysis would not be typically advised due to the before and after data not being independent from each other [64]. Furthermore, due to this association, it is difficult without a control group to establish that the improvements identified in the studies are associated with the intervention rather than other factors [64]. Over time, this review will become less relevant and may possibly become out of date. Thus, it is important to take note of the date of the search strategy when interpreting the findings from this review. However, within the last decade from the point of the search strategy, only three papers have been published.

### Recommendations to future research

The current evidence-base is at a point where no specific clinical recommendations can be made. However, there is enough evidence to suggest that further research is warranted.

Initial studies in this area have been mainly undertaken within hospital and laboratory settings and little is known regarding the use of heat or cold therapy outside these environments. Therefore, until a greater safety profile within these more controlled environments is established, home-based studies are not recommended. Most studies included in the review explored the use of heat therapy for lower limb lymphoedema and there is less certainty regarding its effect on upper limb lymphoedema. Subsequently, there is a requirement for future research to explore the effect of heat on this area. Due to the poor reporting standards within the included studies, it was difficult to establish standardisation of approaches used. Future studies should report greater detail regarding the exact intervention delivered indicating the modalities used, frequency, intensity, and duration of treatment [65,66]. Similarly, greater consistency and transparency is required within outcome reporting. Wherever possible, relevant core outcome sets should be utilized [67]. Future studies exploring the use of cryotherapy with lymphoedema should take a cautionary approach as there is currently little evidence to support the efficacy and safety of this intervention.

## Conclusions

The current evidence-base suggests that heat therapy may help in reducing limb circumference and limb volume when provided over a long period of time (1200–3600 min) at a specific skin temperature (39–42 °C) in a controlled environment (laboratory/hospital/outpatients) in lower limb lymphoedema. When applied within these parameters, there was no evidence that heat therapy was unsafe for patients with lymphoedema. For the use of cold therapy for lymphoedema, there was limited evidence in both effectiveness and safety. Due to the lack of high-quality evidence, no recommendations to practice can be made at this time for the use of both hot and cold therapy for patients with lymphoedema. Further high-quality RCTs are required to explore the effects of heat therapy on people with lymphoedema, with a particular focus on upper limb lymphoedema, adverse events, effects of different heat modalities, intensities, and duration.

## Supplementary Material

Supplemental Material

Supplemental Material

## Data Availability

The data that support the findings of this study are available on request from JEHill1@uclan.ac.uk.
