# Supplementary material for: The effectiveness and safety of heat/cold therapy in adults with lymphoedema: systematic review
Source: Disabil Rehabil. 2023 Jul 10;46(11):2184–95. doi: 10.1080/09638288.2023.2231842 (PMC11147455; doi:10.1080/09638288.2023.2231842)
Supplement: Supplemental Material [file IDRE_A_2231842_SM8594.docx]

**Database: Ovid MEDLINE(R) ALL <1946 to March 21, 2023>**

Date searched: 22/03/2023

| 1 | exp Lymphedema/ |
| --- | --- |
| 2 | (Lymphedema or Lymphoedema or Lymphatic edema or Lymphatic oedema or Lymph static edema or Lymph static oedema).ti,ab,kw,kf. |
| 3 | 1 or 2 |
| 4 | cold temperature/ or freezing/ or hot temperature/ or Body Temperature/ |
| 5 | exp Cryotherapy/ |
| 6 | (ice or cool* or cold or cryotherap* or cryostimulation or cold therap* or cold water immersion or cold pack* or ice massage or psychotherapy or frigotherapy or heat* or heat therap* or heat treatment* or hot or warm* or thermal therapy or thermotherapy or hyperthermia or hot temperature* or hot water immersion or hot pack* or infrared or infra-red or poultice or spa or spas or sauna* or shower* or bath or baths or steam* or microwave or low-frequency low-intensity electrotherapy or core heat load or cool reflex or thermoneutral or hot-tub* or cutaneous cool reflex or Ultrasound therapy).ti,ab,kw,kf. |
| 7 | or/4-6 |
| 8 | 3 and 7 |

**Database: Embase (Ovid) <1974 to 2023 March 21>**

Date searched: 22/03/2023

| 1 | exp lymphedema/ |
| --- | --- |
| 2 | (Lymphedema or Lymphoedema or Lymphatic edema or Lymphatic oedema or Lymph static edema or Lymph static oedema).ti,ab,kw,kf. |
| 3 | 1 or 2 |
| 4 | cold/ |
| 5 | high temperature/ |
| 6 | body temperature/ |
| 7 | cryotherapy/ |
| 8 | exp thermotherapy/ |
| 9 | (ice or cool* or cold or cryotherap* or cryostimulation or cold therap* or cold water immersion or cold pack* or ice massage or psychotherapy or frigotherapy or heat* or heat therap* or heat treatment* or hot or warm* or thermal therapy or thermotherapy or hyperthermia or hot temperature* or hot water immersion or hot pack* or infrared or infra-red or poultice or spa or spas or sauna* or shower* or bath or baths or steam* or microwave or low-frequency low-intensity electrotherapy or core heat load or cool reflex or thermoneutral or hot-tub* or cutaneous cool reflex or Ultrasound therapy).ti,ab,kw,kf. |
| 10 | or/4-9 |
| 11 | 3 and 10 |

**Database: CINAHL Ultimate (EBSCOhost)**

Date searched: 22/03/2023

| S9 | S3 AND S8 |
| --- | --- |
| S8 | S4 OR S5 OR S6 OR S7 |
| S7 | TI ( ice or cool* or cold or cryotherap* or cryostimulation or "cold therap*" or "cold water immersion" or "cold pack*" or "ice massage" or psychotherapy or frigotherapy or heat* or "heat therap*" or "heat treatment*" or hot or warm* or "thermal therapy" or thermotherapy or hyperthermia or "hot temperature*" or "hot water immersion" or "hot pack*" or infrared or "infra-red" or poultice or spa or spas or sauna* or shower* or bath or baths or steam* or microwave or "low-frequency low-intensity electrotherapy" or "core heat load" or "cool reflex" or thermoneutral or "hot-tub*" or "cutaneous cool reflex" or "Ultrasound therapy" ) OR AB ( ice or cool* or cold or cryotherap* or cryostimulation or "cold therap*" or "cold water immersion" or "cold pack*" or "ice massage" or psychotherapy or frigotherapy or heat* or "heat therap*" or "heat treatment*" or hot or warm* or "thermal therapy" or thermotherapy or hyperthermia or "hot temperature*" or "hot water immersion" or "hot pack*" or infrared or "infra-red" or poultice or spa or spas or sauna* or shower* or bath or baths or steam* or microwave or "low-frequency low-intensity electrotherapy" or "core heat load" or "cool reflex" or thermoneutral or "hot-tub*" or "cutaneous cool reflex" or "Ultrasound therapy" ) OR SU ( ice or cool* or cold or cryotherap* or cryostimulation or "cold therap*" or "cold water immersion" or "cold pack*" or "ice massage" or psychotherapy or frigotherapy or heat* or "heat therap*" or "heat treatment*" or hot or warm* or "thermal therapy" or thermotherapy or hyperthermia or "hot temperature*" or "hot water immersion" or "hot pack*" or infrared or "infra-red" or poultice or spa or spas or sauna* or shower* or bath or baths or steam* or microwave or "low-frequency low-intensity electrotherapy" or "core heat load" or "cool reflex" or thermoneutral or "hot-tub*" or "cutaneous cool reflex" or "Ultrasound therapy" ) |
| S6 | (MH "Body Temperature") OR (MH "Heat-Cold Application") |
| S5 | (MH "Hyperthermia, Induced+") |
| S4 | (MH "Cryotherapy") |
| S3 | S1 OR S2 |
| S2 | TI ( Lymphedema or Lymphoedema or "Lymphatic edema" or "Lymphatic oedema" or "Lymph static edema" or "Lymph static oedema" ) OR AB ( Lymphedema or Lymphoedema or "Lymphatic edema" or "Lymphatic oedema" or "Lymph static edema" or "Lymph static oedema" ) OR SU ( Lymphedema or Lymphoedema or "Lymphatic edema" or "Lymphatic oedema" or "Lymph static edema" or "Lymph static oedema" ) |
| S1 | (MH "Lymphedema+") |

**Database: Allied and Complementary Medicine Database (EBSCOhost)**

Date searched: 22/03/2023

| S7 | S3 AND S6 |
| --- | --- |
| S6 | S4 OR S5 |
| S5 | TI ( ice or cool* or cold or cryotherap* or cryostimulation or "cold therap*" or "cold water immersion" or "cold pack*" or "ice massage" or psychotherapy or frigotherapy or heat* or "heat therap*" or "heat treatment*" or hot or warm* or "thermal therapy" or thermotherapy or hyperthermia or "hot temperature*" or "hot water immersion" or "hot pack*" or infrared or "infra-red" or poultice or spa or spas or sauna* or shower* or bath or baths or steam* or microwave or "low-frequency low-intensity electrotherapy" or "core heat load" or "cool reflex" or thermoneutral or "hot-tub*" or "cutaneous cool reflex" or "Ultrasound therapy" ) OR AB ( ice or cool* or cold or cryotherap* or cryostimulation or "cold therap*" or "cold water immersion" or "cold pack*" or "ice massage" or psychotherapy or frigotherapy or heat* or "heat therap*" or "heat treatment*" or hot or warm* or "thermal therapy" or thermotherapy or hyperthermia or "hot temperature*" or "hot water immersion" or "hot pack*" or infrared or "infra-red" or poultice or spa or spas or sauna* or shower* or bath or baths or steam* or microwave or "low-frequency low-intensity electrotherapy" or "core heat load" or "cool reflex" or thermoneutral or "hot-tub*" or "cutaneous cool reflex" or "Ultrasound therapy" ) OR SU ( ice or cool* or cold or cryotherap* or cryostimulation or "cold therap*" or "cold water immersion" or "cold pack*" or "ice massage" or psychotherapy or frigotherapy or heat* or "heat therap*" or "heat treatment*" or hot or warm* or "thermal therapy" or thermotherapy or hyperthermia or "hot temperature*" or "hot water immersion" or "hot pack*" or infrared or "infra-red" or poultice or spa or spas or sauna* or shower* or bath or baths or steam* or microwave or "low-frequency low-intensity electrotherapy" or "core heat load" or "cool reflex" or thermoneutral or "hot-tub*" or "cutaneous cool reflex" or "Ultrasound therapy" ) |
| S4 | (((((ZU "heat") or (ZU "heat therapy")) or ((ZU "cold") or (ZU "cold therapy"))) or ((ZU "cryotherapy"))) or ((ZU "hyperthermia induced"))) or ((ZU "body temperature")) |
| S3 | S1 OR S2 |
| S2 | TI ( Lymphedema or Lymphoedema or "Lymphatic edema" or "Lymphatic oedema" or "Lymph static edema" or "Lymph static oedema" ) OR AB ( Lymphedema or Lymphoedema or "Lymphatic edema" or "Lymphatic oedema" or "Lymph static edema" or "Lymph static oedema" ) OR SU ( Lymphedema or Lymphoedema or "Lymphatic edema" or "Lymphatic oedema" or "Lymph static edema" or "Lymph static oedema" ) |
| S1 | (ZU "lymphedema") |

**Database: Cochrane Library via Wiley (all databases)**

Date searched: 22/03/2023

| #1 | MeSH descriptor: [Lymphedema] explode all trees |
| --- | --- |
| #2 | (Lymphedema or Lymphoedema or "Lymphatic edema" or "Lymphatic oedema" or "Lymph static edema" or "Lymph static oedema"):ti,ab,kw |
| #3 | #1 OR #2 |
| #4 | MeSH descriptor: [Cold Temperature] explode all trees |
| #5 | MeSH descriptor: [Freezing] explode all trees |
| #6 | MeSH descriptor: [Hot Temperature] explode all trees |
| #7 | MeSH descriptor: [Body Temperature] explode all trees |
| #8 | MeSH descriptor: [Cryotherapy] explode all trees |
| #9 | (ice or cool* or cold or cryotherap* or cryostimulation or cold therap* or "cold water immersion" or cold pack* or "ice massage" or psychotherapy or frigotherapy or heat* or heat therap* or heat treatment* or hot or warm* or "thermal therapy" or thermotherapy or hyperthermia or hot temperature* or "hot water immersion" or hot pack* or infrared or "infra-red" or poultice or spa or spas or sauna* or shower* or bath or baths or steam* or microwave or "low-frequency low-intensity electrotherapy" or "core heat load" or "cool reflex" or thermoneutral or hot-tub* or "cutaneous cool reflex" or "Ultrasound therapy"):ti,ab,kw |
| #10 | {OR #4-#9} |
| #11 | #3 AND #10 |

**Database: Web of Science: (Science Citation Index Expanded (SCI-EXPANDED)--1970-present; Social Sciences Citation Index (SSCI)--1970-present; Arts & Humanities Citation Index (AHCI)--1975-present; Conference Proceedings Citation Index – Science (CPCI-S)--1990-present; Conference Proceedings Citation Index – Social Science & Humanities (CPCI-SSH)--1990-present; Emerging Sources Citation Index (ESCI)--2017-present)**

Date searched: 22/03/2023

#1 TS=(Lymphedema or Lymphoedema or ""Lymphatic edema"" or ""Lymphatic oedema"" or ""Lymph static edema"" or ""Lymph static oedema"")"

#2 TS=(ice or cool* or cold or cryotherap* or cryostimulation or "cold therap*" or "cold water immersion" or "cold pack*" or "ice massage" or psychotherapy or frigotherapy or heat* or "heat therap*" or "heat treatment*" or hot or warm* or "thermal therapy" or thermotherapy or hyperthermia or "hot temperature*" or "hot water immersion" or "hot pack*" or infrared or "infra-red" or poultice or spa or spas or sauna* or shower* or bath or baths or steam* or microwave or "low-frequency low-intensity electrotherapy" or "core heat load" or "cool reflex" or thermoneutral or "hot-tub*" or "cutaneous cool reflex" or "Ultrasound therapy")

#3 #1 AND #2
