# Supplementary material for: The effectiveness and safety of heat/cold therapy in adults with lymphoedema: systematic review
Source: Disabil Rehabil. 2023 Jul 10;46(11):2184–95. doi: 10.1080/09638288.2023.2231842 (PMC11147455; doi:10.1080/09638288.2023.2231842)
Supplement: Supplemental Material [file IDRE_A_2231842_SM8591.docx]

Appendix 1: Search strategy for MEDLINE

| Search Number | Query | Hits | Database | Date searched |
| --- | --- | --- | --- | --- |
| 1 | exp Lymphedema/ | 13190 | Ovid MEDLINE(R) and Epub Ahead of Print, In-Process, In-Data-Review & Other Non-Indexed Citations and Daily 1946 to January 27, 2022 | 28/01/2022 |
| 2 | (Lymphedema or Lymphoedema or Lymphatic edema or Lymphatic oedema or Lymph static edema or Lymph static oedema).ti,ab,kw,kf. | 11509 |  |  |
| 3 | 1 or 2 | 17112 |  |  |
| 4 | cold temperature/ or freezing/ or hot temperature/ or Body Temperature/ | 233437 |  |  |
| 5 | exp Cryotherapy/ | 26784 |  |  |
| 6 | (ice or cool* or cold or cryotherap* or cryostimulation or cold therap* or cold water immersion or cold pack* or ice massage or psychotherapy or frigotherapy or heat* or heat therap* or heat treatment* or hot or warm* or thermal therapy or thermotherapy or hyperthermia or hot temperature* or hot water immersion or hot pack* or infrared or infra-red or poultice or spa or spas or sauna* or shower* or bath or baths or steam* or microwave or low-frequency low-intensity electrotherapy or core heat load or cool reflex or thermoneutral or hot-tub* or cutaneous cool reflex or Ultrasound therapy).ti,ab,kw,kf. | 920986 |  |  |
| 7 | or/4-6 | 1050148 |  |  |
| 8 | 3 and 7 | 367 |  |  |
